# EMBEDDING EVIDENCE-BASED CAREGIVER SUPPORT IN COMMUNITY-BASED PROGRAMS: THE ADS PLUS PROGRAM

**DOI:** 10.1093/geroni/igad104.0521

**Published:** 2023-12-21

**Authors:** Laura Gitlin, Joseph Gaugler, Kenneth Hepburn

## Abstract

Adult Day Services (ADS) are a community-based option in long-term care, providing a safe, interactive, and managed environment for people with dementia (PWD). ADS also serve racially/ethnically diverse communities, provide respite for family caregivers and help people age-in-place. Nevertheless, research reveals inconsistent benefits of ADS for caregivers of clients and ADS do not typically offer evidence-based caregiver programs as part of routine care. This symposium reports on the results of a novel NIA funded multi-site Hybrid Type I trial with pragmatic elements which evaluated the effectiveness of embedding a caregiver support program, ADS Plus, in ADS and delivered by staff. ADS Plus, provides caregivers with dementia education, stress reduction techniques, validation/support, referrals and linkages, and strategies to address caregiver-identified challenges over 12-months. Reported are the quantitative and cost outcomes, implementation processes, and challenges and lessons learned. Dr. Gitlin will present the main quantitative study outcomes of this multi-site trial involving 34 sites nationally and 234 caregivers, which demonstrated benefits for caregivers and ADS. Next, Dr. Marx will examine implementation findings including session completion and content. Dr. Gaugler will present findings from the qualitative arm examining perceived benefits of ADS Plus by staff and participants. Ms. Prioli will then examine cost savings associated with ADS Plus whereas Dr. Parker will address barriers when partnering with home and community-based services including developing and maintaining partnerships, institutional review board hurdles and staff readiness. To conclude, our discussant, Dr. Kenneth Hepburn, will offer perspectives on moving evidence-based programs into community-based settings such as ADS. This is a Behavioral Interventions for Older Adults Interest Group Sponsored Symposium.

